# Maternal Age at First Delivery Is Associated with the Risk of Metabolic Syndrome in Postmenopausal Women: From 2008–2010 Korean National Health and Nutrition Examination Survey

**DOI:** 10.1371/journal.pone.0127860

**Published:** 2015-05-26

**Authors:** Jeong Han Sim, Dawn Chung, Jung Soo Lim, Mi Young Lee, Choon Hee Chung, Jang Yel Shin, Ji Hye Huh

**Affiliations:** 1 Department of Internal Medicine, Yonsei University, Wonju College of Medicine, Wonju, Gwangwondo, Korea; 2 Department of Obstetrics and Gynecology Yonsei University, Wonju College of Medicine, Wonju, Gwangwondo, Korea; Medical University Innsbruck, AUSTRIA

## Abstract

**Background:**

Recent cross-sectional studies demonstrated that earlier maternal age at first childbirth is correlated with a higher risk of diabetes in postmenopausal women. In this study, we evaluated whether the age at first delivery is associated with the risk of metabolic syndrome (MetS) in postmenopausal women.

**Methods:**

A total of 4,261 postmenopausal women aged 45 years or older were analyzed using data generated from Korea National Health and Nutrition Examination Surveys (2008–2010). Subjects were divided into three groups according to the maternal age at first delivery as follows: ≤ 20 years (n=878), 21-25 years (n=2314), and ≥ 26 years (n=1069).

**Results:**

Approximately 37% of subjects had MetS. The prevalence of MetS showed a gradual increase as maternal age at first delivery decreased (≥ 26 years = 30.9% vs. 21-25 years = 39.9% vs. ≤ 20 years = 50.8%, respectively, p < 0.001). Central obesity indices such as trunk fat mass and waist circumference were significantly higher in the group aged ≤ 20 years than other groups. After adjustments for confounding factors, the odds ratios (ORs) for predicting the presence of MetS increased gradually as first delivery age decreased (≥ 26 years vs. 21-25 years vs. ≤ 20 years: OR [95% CI] = 1 vs. 1.324 [1.118-1.567] vs. 1.641 [1.322-2.036], respectively). Among components of MetS, younger maternal age at first delivery (≤ 20 years) was significantly associated with increased waist circumference (OR [95% CI] = 1.735 [1.41-2.13]), elevated blood pressure (1.261 [1.02-1.57]), high triglyceride (1.333 [1.072-1.659]), and low HDL-cholesterol (1.335[1.084-1.643]).

**Conclusions:**

Our findings suggest that younger maternal age at first delivery is independently associated with a higher risk of central obesity and MetS in postmenopausal women.

## Introduction

Metabolic syndrome (MetS) is characterized by several metabolic abnormalities such as dysglycemia, central obesity, dyslipidemia and hypertension [1]. It is known to be associated with increased cardiovascular disease and all-cause mortality in the general population [2]. As the prevalence of MetS has increased globally, it has become a major health problem, especially in postmenopausal women who are at high risk for metabolic abnormalities. Thus, in postmenopausal women, it is important to identify subjects who have high risk factors for MetS in earlier life.

To date, several studies have suggested that reproductive factors in earlier life are related to health problems in later life [3, 4]. Early age at menarche was associated with metabolic syndrome [4], type 2 diabetes [5, 6], cardiovascular disease and overall mortality [7] in elderly women. Higher parity or gravidity was associated with metabolic syndrome in older Chinese women [8] and type 2 diabetes patients [9]. Recently, younger age at first delivery was also considered to be a risk factor for various metabolic disorders in adult life because early pregnancy may alter overall metabolism of individual, for example by inducing insulin resistance. Kim et al. reported that adolescent pregnancy was independently associated with a higher risk of type 2 diabetes in postmenopausal women [10]. Moreover, another study demonstrated that first delivery at young age was related to subsequent development of cardiovascular disease, especially myocardial infarction [11].

Although adolescent pregnancy is an emerging risk factor for metabolic disorders, few studies have examined the association between maternal age at first delivery and the presence of MetS in later life. In addition, while some studies have already suggested that the association between maternal age at first delivery and metabolic disorders is due to the development of obesity in adolescent pregnancy, no studies have compared body composition indices in postmenopausal women according to their age at first delivery. Therefore, the aim of our study was to determine whether the maternal age at first delivery is associated with MetS and its components in postmenopausal women. We also evaluated the relationship between maternal age at first delivery and body composition indices measured by dual-energy X-ray absorptiometry, which is known to be a gold standard for measuring fat and muscle mass in older populations [12].

## Materials and Methods

### Study population and design

We recruited participants from 2008–2010 Korea National Health and Nutrition Examination Surveys. The KNHANES has been performed periodically since 1998 by the Division of Chronic Disease Surveillance of the Korean Centers for Disease Control and Prevention in order to assess the health and nutritional status of the civilian, non-institutionalized population of Korea. It was a cross-sectional and nationally representative survey, composed of a health interview survey, a nutrition survey, and a health examination survey. The data were collected by household interviews and by direct, standardized physical examinations conducted in mobile examination centers. Nutritional status and medical history were evaluated using a 24-h recall method. Alcohol consumption was indicated as “yes” for participants who consumed at least two units of alcohol every week over the last year. Regular exercise was indicated as “yes” when the subject exercised for more than 20 min at a time and more than three times per week. Self-reported questionnaires were administered to determine household income, education level and residential area. Residential area was categorized according to the Korean administrative district as an urban or rural area. We recruited 5952 postmenopausal women aged 45 and older. Postmenopausal status was defined as the self-reported cessation of menstruation for more than 1 year only and we excluded women who had undergone hysterectomy (n = 941). Subjects with any pathological disorders (such as current cancer (n = 88), thyroid disorder (n = 193), renal failure (n = 21), or hepatic failure (n = 12)) or subjects taking medications (such as corticosteroids and statin (n = 436)) known to alter metabolic parameters were excluded from the analysis. Finally, a total of 4,261 postmenopausal women aged 45 years or older were analyzed in this study. To investigate the association between maternal age at first delivery and metabolic parameters, these subjects were classified into 3 groups according to their age at first delivery: ≤ 20 years (n = 878), 21–25 years (n = 2314), and ≥ 26 years (n = 1069).

### Ethics Statement

Because the KNHANES IV survey data are publicly available, ethical approval was not required for this study. Prior to the survey, all participants were informed that they had been randomly chosen to participate in the KNHANES IV survey with the right to refuse to be involved in further analyses, and signed informed consents were obtained. The data we used from the KNHANES database were fully anonymized.

### Measurements

Body weight and height were obtained using standard protocols. Waist circumference was measured at the narrowest point between the lower borders of the rib cage and the uppermost borders of the iliac crest at the end of normal expiration. Well-trained observers manually measured blood pressure with a mercury sphygmomanometer (Baumanometer; Baum, Copiague, NY). Body composition including fat mass and appendicular skeletal muscle mass (ASM) were measured by dual-energy X-ray absorptiometry (DXA) (QDR 4500A; Hologic Inc., Waltham, MA) in all participants who met inclusion criteria (n = 4261). Collected blood samples were immediately refrigerated, transported to the Central Testing Institute in Seoul, Korea, and analyzed within 24 h. Fasting plasma glucose, total cholesterol, triglycerides (TG), and high-density lipoprotein (HDL) cholesterol levels were measured with a Hitachi 700–110 chemistry analyzer (Hitachi, Tokyo, Japan). Serum 25(OH)D concentrations were measured by radioimmunoassay (DiaSorin Inc., Stillwater, MN, USA) using a γ-counter (1470 Wizard; PerkinElmer, Turku, Finland). The homeostasis model assessment of insulin resistance (HOMA-IR) was calculated using the following formula: [fasting plasma glucose (milligrams per deciliter) ×fasting insulin (milli-international units per milliliter)]/22.5 [13].

### Definition of MetS

We defined the metabolic syndrome using modified National Cholesterol Education Program Adult Treatment Panel III (NCEP-ATP III) criteria [1] as the presence of 3 or more of the following components: 1) waist circumference ≥ 80 cm in women, adopted from waist circumference cutoff modifications for Asian populations as suggested by the Asia-Pacific guidelines [14]; 2) TG ≥ 150 mg/dL; 3) HDL cholesterol < 50 mg/dL in women; 4) blood pressure ≥ 130/85 mmHg or treatment for hypertension; and 5) fasting glucose level ≥ 100 mg/dL or treatment for diabetes.

### Statistical analyses

Statistical analyses were conducted using PASW Statistics version 18 (SPSS Inc., Chicago, IL, USA). Analysis of covariance (ANCOVA) test adjusted age followed by post hoc analysis using Bonferroni post hoc comparison was used to compare clinical characteristics among the groups. For categorical variables, a Chi square test was used to compare frequencies among the groups. Multiple logistic regression analysis was used to examine the adjusted odds ratios of maternal age at first delivery for metabolic syndrome and its components. Analyses were adjusted for potential confounders including age, current smoking, regular exercise, alcohol intake, number of pregnancies, age at menarche, hormone replacement therapy and total energy intake.

## Results

### Baseline Characteristics of Patients

Table 1 summarizes the demographic and clinical characteristics of the patients classified into three groups by maternal age at first delivery. Because there were differences in age between the groups, we performed ANCOVA test adjusted age to compare characteristics among three groups. Mean age of all patients was 59.71 ± 13.25 years (range 45–98 years). The overall prevalence of MetS in postmenopausal women was 36.84%. The prevalence of MetS showed a gradual increase with decreasing maternal age at first delivery (≥ 26 years = 30.9% vs. 21–25 years = 39.9% vs. ≤ 20 years = 50.8%, respectively, P<0.001). Obesity indices such as body weight, body mass index (BMI), waist circumference, total fat mass and truncal fat mass were higher in the group aged ≤ 20 years compared with those of other groups. In addition, subjects with earlier age at first delivery were more likely to have lower family income and education level and they also tended to be more resident in rural area. They were also tended to have higher carbohydrate and fat intake and lower potassium intake. Fasting glucose, HbA1c, HOMA-IR and TG were also higher in the group aged ≤ 20 years than those in the other groups. Age at menarche and the percentage of hormone replacement therapy was lower in the group aged ≤ 20 years. Number of pregnancies was higher in the group aged ≤ 20 years. Systolic blood pressure (SBP), diastolic blood pressure (DBP), total cholesterol, LDL cholesterol and ASM/weight were not different between the three groups.

**Table 1:** Characteristics of the study population according to the maternal age at first delivery.

	Age at first delivery (years)			
	≤ 20 (n = 878)	21–25 (n = 2314)	≥26 (n = 1069)	P-value
Age ^a^ (year)	69.35±9.98† ‡	63.51±9.00† #	59.77±8.57‡ #	<0.001
Height (cm)	152.70±0.19	153.07±0.11	153.20±0.17	0.133
Weight (Kg)	57.55±0.29‡	56.78±0.17#	55.79±0.26‡ #	<0.001
BMI (kg/m^2^)	24.64±0.11† ‡	24.21±0.07† #	23.74±0.1‡ #	<0.001
Waist circumference (cm)	83.85±0.32† ‡	82.70±0.19† #	80.75±0.29‡ #	<0.001
Current smoking ^a^ (%)	23 (10.7%)† ‡	21(3.6%)†	19(5.4%)‡	<0.001
Regular exercise ^a^ (%)	16 (7.5%)†	70 (11.9%)	50 (14.2%)†	0.055
Family income ^a^				<0.001
Low	458(52.9%)† ‡	866(38.2%)† #	238 (22.6%)‡ #	
Moderate-Low	212 (24.5%)	569 (25.1%)	283 (26.9%)	
Moderate-High	115 (13.3%)‡	416 (18.2%)#	273 (25.9%)‡ #	
High	80 (9.2%)† ‡	418 (18.4%)† #	259 (24.6%)‡ #	
Education ^a^				<0.001
Elementary	785 (90.4%)† ‡	1596 (69.2%)† #	430 (40.4%)‡ #	
Middle school	64 (7.4%)† ‡	332 (14.4%)†	209 (19.6%)‡	
High school	18 (2.1%)† ‡	308 (13.4%)† #	307 (28.8%)‡ #	
College	1 (0.1%)† ‡	70 (3%)† #	119 (11.2%)‡ #	
Residence in rural area ^a^ (%)	392 (44.6%)† ‡	842 (36.4%)† #	220 (20.6%)‡ #	<0.001
Total energy intake (kcal/day)	1521.12±20.84	1569.21±12.38	1534.01±18.69	0.075
Carbohydrate intake (g/day)	287.81±4.02	294.62±2.39	284.72±3.61	0.047
Fat intake (g/day)	20.23±0.61† ‡	21.95±0.36†	22.81±0.54‡	0.008
Sodium intake (mg/day)	3841.27±91.56	3810.67±54.37	3708.93±82.11	0.502
Potassium intake (mg/day)	2498.13±51.66† ‡	2641.42±30.68†	2668.55±46.33‡	0.032
SBP (mmHg)	128.37±0.61	127.21±0.36	127.80±0.55	0.228
DBP (mmHg)	77.94±0.35	77.53±0.21	77.87±0.32	0.601
Fasting glucose	101.98±0.88	101.52±0.51	99.37±0.76	0.038
HbA1c (%)	7.34±0.11	7.37±0.07#	6.96±0.13#	0.016
HOMA-IR	2.98±0.10† ‡	2.65±0.06†	2.50±0.08‡	0.001
Total cholesterol (mg/dL)	202.02±1.35	199.92±0.78	200.51±1.17	0.402
LDL cholesterol (mg/dL)	120.80±1.23	120.53±0.72	121.45±1.07	0.776
HDL cholesterol (mg/dL)	51.09±0.44	51.92±0.26	52.51±0.38	0.063
Triglyceride (mg/dL)	150.66±3.22† ‡	137.36±1.87†	132.78±2.79‡	<0.001
Vitamin D	19.63±0.27‡	19.34±0.15#	18.41±0.23‡ #	0.001
Total fat mass (kg)	19.89±0.19‡	19.52±0.11	19.15±0.16‡	0.014
Truncal fat mass (kg)	10.45±0.11‡	10.32±0.06	9.98±0.08‡	<0.001
ASM/weight (%)	24.68±0.09	24.87±0.06	24.90±0.08	0.181
Age at menarche (years)	15.66±0.07†	16.10±0.04† #	15.84±0.06#	<0.001
Number of pregnancies	6.01±0.17† ‡	5.35±0.1† #	4.47±0.15‡ #	<0.001
Hormone replacement therapy ^a^ (%)	81 (9.2%)† ‡	371 (16.1%)† #	222 (20.8%)‡ #	<0.001
Metabolic syndrome ^a^ (%)	396 (50.8%)† ‡	860 (39.9%)† #	314(30.9%)‡ #	<0.001

Data presented as age-adjusted mean ± standard error or n (%), except for age.

^a^ Non-adjusted values

BMI: body mass index; SBP: systolic blood pressure; DBP: diastolic blood pressure; HbA1c: Hemoglobin A1c; HOMA-IR: homeostasis model assessment-insulin resistance; LDL: low-density lipoprotein; HDL: high-density lipoprotein; ASM: appendicular skeletal mass

^†^: The difference between ≤ 20 and 21–25: p<0.05 after post hoc comparison (Bonferroni test for continuous variables)

^‡^: The difference between ≤ 20 and ≥26: p<0.05 after post hoc comparison (Bonferroni test for continuous variables)

^#^: The difference between 21–25 and ≥26: p<0.05 after post hoc comparison (Bonferroni test for continuous variables)

### Differences in Obesity Indices between groups by age at first delivery

**Fig 1:**
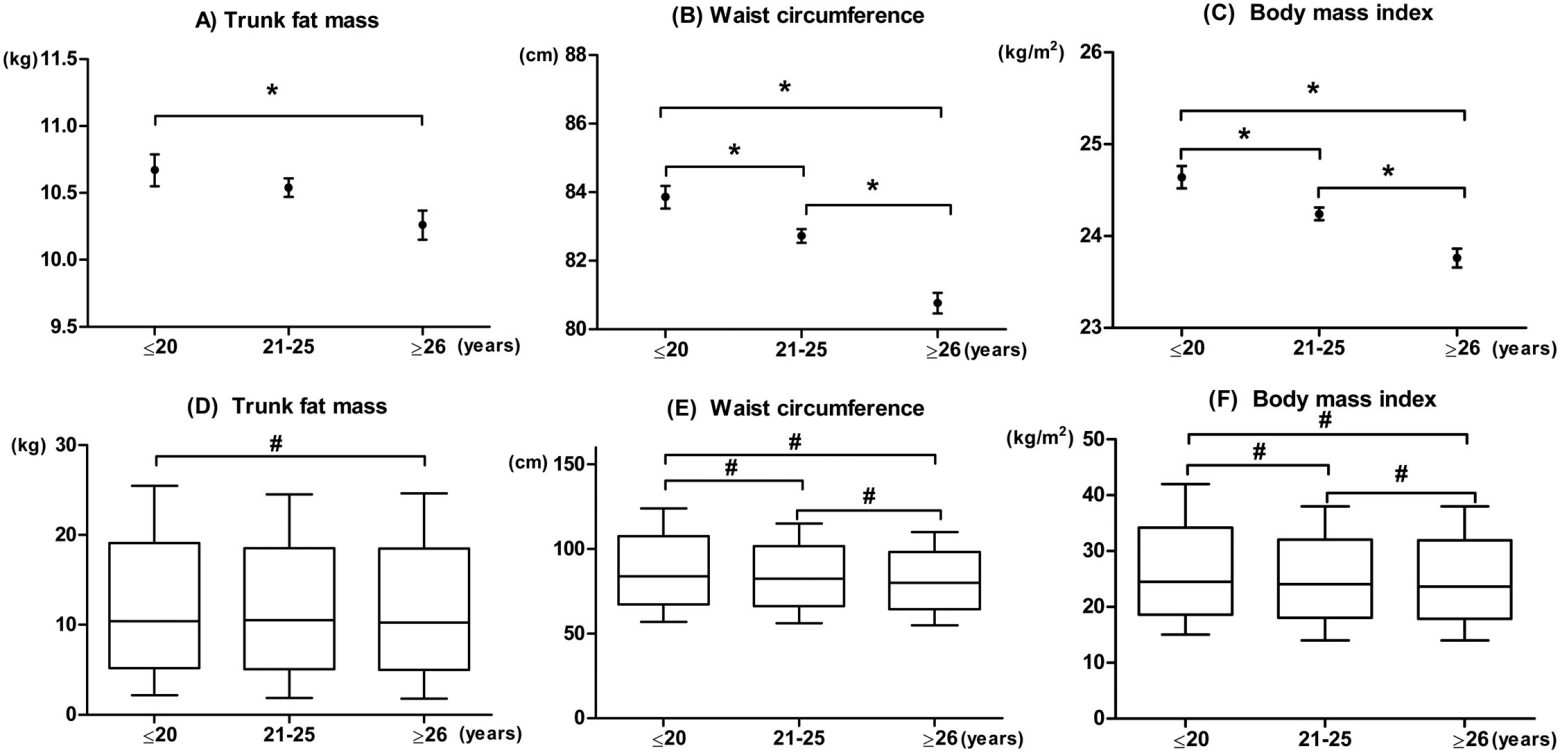
Adjusted mean trunk fat mass (A), waist circumference (B) and body mass index (BMI) (C) in the different age at first delivery groups. Data are expressed as estimated marginal mean and standard error (*, P<0.05 by ANCOVA after controlling for adjusted for age, number of pregnancies, age at menarche, total energy intake, regular exercise and hormone replacement therapy with Bonferroni correction). Box and whisker plots of the trunk fat mass (D), waist circumference (E) and BMI (F) among different age at first delivery groups. Boxes show interquartile range with median indicated, and the whiskers indicate the minimum and maximum value (#, P<0.05 by ANOVA with Bonferroni correction).

To examine the independent relationship between age at first delivery and obesity indices, especially central obesity indices, ANCOVA models were constructed with age, number of pregnancies, age at menarche, total energy intake, regular exercise and hormone replacement therapy added as covariates (Fig 1A–1C). As shown in Fig 1A, trunk fat mass was significantly higher in the group aged ≤ 20 years compared with those of group aged ≥ 26 years. Waist circumference and BMI were also significantly higher in the group aged ≤ 20 years than in other groups (Fig 1B and 1C). Total body fat mass was also higher in the group aged ≤ 20 years compared with other groups (≤ 20 years = 19.84 ± 0.2 kg vs. 21–25 years = 19.58 ± 0.11 kg vs. ≥ 26 years = 19.16 ± 0.17kg, respectively, P = 0.027). Fig 1D–1F show box and whisker plots of the trunk fat mass (D), waist circumference (E) and BMI (F) among different age at first delivery groups. Similarly, trunk fat mass, waist circumference and BMI were significantly higher in the group aged ≤ 20 years mainly due to the higher maximum value, with only a small increase in the minimum value when compared with the other groups.

### Prevalence of MetS according to age at first delivery

**Fig 2:**
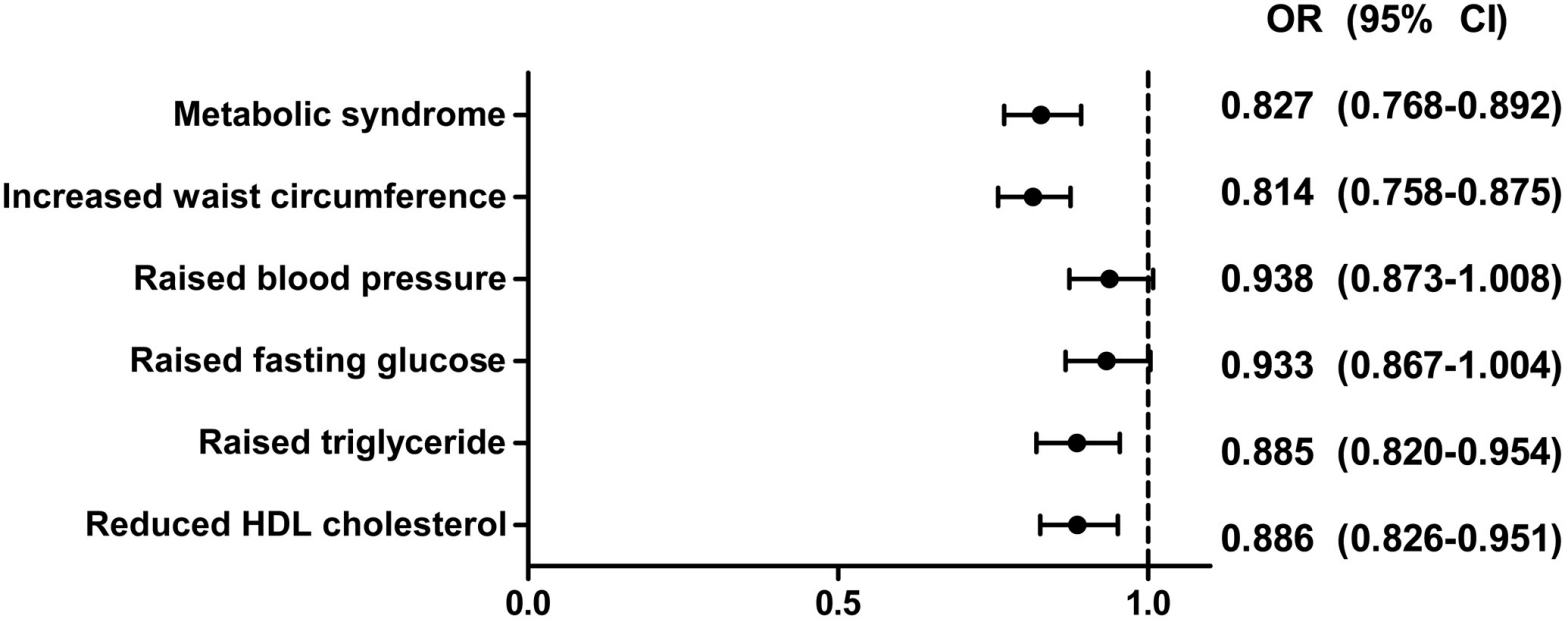
Adjusted odds ratios (ORs) with 95% confidence interval (CI) of metabolic syndrome and its components for each standard deviation (SD) increase in maternal age at first delivery. Models were adjusted for age, current smoking, regular exercise, alcohol intake, number of pregnancies, age at menarche, hormone replacement therapy and total energy intake.

After adjusting for age, current smoking, alcohol drinking, physical activity, total energy intake, parity, age at menarche, and hormone replacement therapy, the odds ratios (OR) for predicting the presence of MetS increased gradually as first delivery age decreased (≥ 26 years vs. 21–25 years vs. ≤ 20 years: OR [95% CI] = 1 vs. 1.324 [1.118–1.567] vs. 1.641 [1.322–2.036], respectively) (Table 2). Among the components of MetS, the group aged ≤ 20 years was significantly associated with increased waist circumference (OR = 1.735 [95% CI = 1.411−2.133]), raised blood pressure (OR = 1.261 [95% CI = 1.015–1.56]), raised TG (OR = 1.333 [95% CI = 1.072–1.659]) and reduced HDL cholesterol (OR = 1.333 [95% CI = 1.084–1.643]). The group aged 21–25 years was significantly associated with only increased waist circumference (OR = 1.513 [95% CI = 1.285−1.782]). Each standard deviation (SD) increase in maternal age at first delivery was associated with a 17.3% reduction in metabolic syndrome (Fig 2). Among components of MetS, each SD increase in maternal age at first delivery was significantly associated with an 18.6% reduction in increased waist circumference, an 11.5% reduction in raised TG and an 11.4% reduction in reduced HDL cholesterol.

**Table 2:** Adjusted odds ratios (ORs) with 95% confidence interval (CI) of metabolic syndrome and its components according to age at first delivery.

	Age at first delivery		
	≤20 years	→21–25years	≥26years
Metabolic syndrome	1.641 (1.322–2.036)	1.324(1.118–1.567)	reference
Increased waist circumference	1.735(1.411–2.133)	1.513(1.285–1.782)	reference
Raised Blood Pressure	1.261(1.015–1.568)	1.101(0.938–1.292)	reference
Raised Fasting Glucose	1.181(0.953–1.464)	1.089(0.929–1.298)	reference
Raised Triglyceride	1.333(1.072–1.659)	1.071(0.902–1.271)	reference
Reduced HDL cholesterol	1.335(1.084–1.643)	1.145(0.976–1.324)	reference

Adjusted for age, current smoking, regular exercise, alcohol intake, number of pregnancies, age at menarche, hormone replacement therapy and daily total energy intake.

## Discussion

The present study found that adolescent pregnancy (maternal age at first delivery ≤ 20 years) was significantly associated with a higher risk of MetS in postmenopausal women. Furthermore, we demonstrated that earlier age at first delivery was intimately associated with central obesity and central obesity-related factors such as increase in TG and decrease in HDL cholesterol. To the best of our knowledge, this is the first population-based study to represent the association between maternal age at first delivery and metabolic parameters including body composition measured by DXA.

Pregnancy is characterized by an increase in insulin resistance due to the increase of cortisol, progesterone, human placental lactogen, and tumor necrosis factor α and the decrease of adiponectin [15]. Pregnancy also causes an increase in plasma volume due to the activation of the renin angiotensin system [16] and the creation of a thrombophilic state, which leads to the progression of atherosclerosis [17]. Several studies have demonstrated that increasing parities were independently associated with an increased prevalence of MetS [18], cardiovascular events [19] and systemic arterial remodeling [20]. Therefore, childbearing has been suggested to have long-lasting effects on the metabolic and cardiovascular system in individuals. From this background, we speculated that the timing of first childbearing may also influence metabolic disorders in later life. Because there is a paucity of systemic data for the association between age at first delivery and MetS, we attempted to investigate this association in community-dwelling Korean postmenopausal women.

In our study, we observed a prominent association between maternal age at first delivery and MetS. The possible link between maternal age at first delivery and metabolic disorders can be explained by the following mechanisms. The first possible mechanism is that adolescent pregnancy may affect the development of obesity. Pregnancy is characterized by body fat accumulation, associated with both hyperphagia and increased lipogenesis [21]. Therefore, obesity induced by pregnancy at young age may have a long-lasting effect on body composition in later life and consequently it may increase metabolic disease risk. Actually, in our study, adolescent pregnancy (age at first delivery ≤ 20) was independently associated with increased obesity indices. And we also presented that adolescent pregnancy was associated with obesity related metabolic disorders such as raised blood pressure, raised TG and reduced HDL cholesterol. Secondly, because adolescents have relatively immature organs, the physiologic changes of pregnancy such as increase in insulin resistance due to increase of inflammatory cytokines may deleteriously influence the normal development of organs [22]. As a result, organs related to metabolism may not work properly; this may cause metabolic problems in later life. Besides, early exposure to high doses of estrogen also may have a negative effect on the metabolism, just like the known effect of early menarche on metabolic disorders [7, 23]. Finally, in our study, there were significant differences in socioeconomic factors such as household income, education level and residential area among different age at first delivery groups. In addition, we also observed that subjects with earlier age at delivery tended to have higher carbohydrate and fat intake and lower potassium intake. Therefore, lower socioeconomic status and poor quality of dietary pattern in subjects with earlier age at delivery may exert a bad influence on their metabolic parameters.

The major findings of our study include the strong association between maternal age at first delivery and obesity indices, especially central obesity. Recently, there are growing evidences obesity, especially central obesity of greater importance metabolically than that of overall obesity because intra-abdominal (visceral) fat is associated with obesity-related insulin resistance, cardiovascular disease, lower HDL cholesterol levels, and progression to type 2 diabetes, particularly among women [24]. Increased release of free fatty acids from visceral fat into the portal vein might play a major role in the development of insulin resistance in central obesity [25]. In this context, we demonstrated that earlier age at first delivery was significantly associated with increase in waist circumference, truncal fat mass as well as body weight, BMI and total fat mass as directly measured by DXA even after adjustment for confounding factors. A similar tendency was observed in other previous studies [26, 27]. Wen et al. reported that the later the first birth occurs, the lower the postmenopausal amount of body fat will be [26]. Kirchengast et al. also presented a significant association between age at first childbirth and body fat after menopause [27]. However, these studies analyzed only total body fatness and they did not show a direct association between age at first birth and central obesity indices measured by DXA like our study. Although the exact mechanism for this association remains to be definitively proven, we can assume from our results that adolescent pregnancy might contribute to the deleterious of body compositions and it may lead to the development of future metabolic disorders in later life. Further investigation is needed to determine whether earlier age of first delivery is associated with greater increases visceral fat deposition and whether the adverse effects of earlier age of first delivery on metabolic risk profiles in elderly women are related to increases in visceral adipose tissue levels.

The major strength of this study lies in that the data were collected from a nationwide survey that included 4,261 postmenopausal women throughout Korea. This is the first population-based study that extensively investigated the potential impact of early pregnancy on metabolic profiles and body composition in later life. Nevertheless, our study also has some limitations. First, as the present study was a cross-sectional study and not a longitudinal study, a causal relationship between maternal age at first delivery and metabolic syndrome could not be definitively established. In addition, because the time interval from the first childbirth to the point of survey was too long, other unexpected factors may have influenced the metabolic parameters of later life. And, because we could not collect other early-life environmental conditions besides age at first delivery, we could not adjust for other early-life environmental conditions. Third, misclassification of maternal age at first delivery might have happened due to the retrospective assessment of this variable. Fourth, this cohort study did not collect information about individual’s pregnancy complications such as gestational diabetes or preeclampsia which may impact on the development of metabolic disorders.

In conclusion, our study is the largest population-based study to examine the association between maternal age at first delivery and metabolic components including body composition. Adolescent pregnancy and delivery were closely associated with increased incidence of metabolic syndrome in later life. Especially, maternal age at first delivery showed a close relationship with indices of central obesity measured by DXA. These findings suggest that a history taking for adolescent pregnancy may help to identify women at higher risk for MetS and central obesity.
